# Epidemiological study of canine parvovirus infection in and around Bhubaneswar, Odisha, India

**DOI:** 10.14202/vetworld.2015.33-37

**Published:** 2015-01-09

**Authors:** Monalisa Behera, S. K. Panda, P. K. Sahoo, A. P. Acharya, R. C. Patra, Sweta Das, S. Pati

**Affiliations:** 1 Department of Veterinary Pathology, College of Veterinary Science and Animal Husbandry, Orissa University of Agriculture and Technology, Bhubaneswar, Odisha, India; 2 Fish Health Management Division, Central Institute of Freshwater Aquaculture, Kausalyaganga, Bhubaneswar, Odisha, India; 3 Department of Veterinary Medicine, College of Veterinary Science and Animal Husbandry, Orissa University of Agriculture and Technology, Bhubaneswar, Odisha, India

**Keywords:** canine parvovirus, epidemiology, polymerase chain reaction

## Abstract

**Aim:**

An epidemiological study of canine parvovirus infection in dogs in and around Bhubaneswar, Odisha was conducted between December 2012 to March 2013 and prevalence rate was studied on the basis of age, breed, and sex.

**Materials and Methods:**

A total of 71 fecal samples from suspected diarrheic dogs were collected in sterile phosphate buffer saline (10% W/V) and examined by polymerase chain reaction (PCR) for detection of canine parvo virus infection, followed by epidemiological study in relation to age, breed, and sex.

**Results:**

Of 71 samples analyzed, 29 (40.85%) were found to be positive by PCR assay. The infection was higher in Deshi/local breeds (34.48%), followed by German shepherd (17.24%), equal incidences in mixed and Labrador retriever (10.34%), Rottweiler and German spitz showed 6.90% each and finally lower incidences in four breeds (3.45%) such as Dalmatians, Nea politan mastiff, Pug and Great Dane. Age-wise prevalence study revealed the infection being more in the age group of 3-6 months (41.37%), followed by equal incidences of 27.59% in 1-3 months and 6-12 months age group, and a low incidence in age groups above 12 months (3.45%). The incidence was predominantly higher in males (86.21%) than females (13.79%).

**Conclusions:**

The epidemiological analysis revealed that the breed wise prevalence was found to be more in Deshi breeds as compared to others, age groups below 6 months were found to be more prone to parvovirus infection and males were mostly infected.

## Introduction

Dog has become indispensable in man’s life as an embodiment of love and affection. Dogs have diversified utility ranging from tracking, hunting, instruments of war, bomb detecting squad and healer of both physical and emotional problems of humans, detecting criminals and guiding blind people [1]. In view of the need of dogs in these above aspects it is necessary to study the various diseases that are producing a high morbidity and case fatality rate. Among the viral diseases the predominantly occurring diseases includes canine parvoviral infection, canine distemper, corona virus infection, canine hepatitis, canine parainfluenza and rabies [2].

Canine parvo virus (CPV) infection is a highly infectious viral disease of dogs of great concern to pet owners, practicing veterinarians and scientists due to its high morbidity and mortality rates. Parvo virus infects dogs of all age groups, but puppies are most severely affected than adults [2]. Parvo virus infection is most commonly manifested with signs like, vomiting, bloody diarrhea and severe leukopenia. Outbreaks of CPV have been reported from many countries including India. The prevalence study in India was first reported by Balu and Thangaraj [3] in Madras. The pattern of disease experienced in a population is largely influenced by the susceptibility of host, environmental conditions such as housing, hygiene, population density, and pathogenicity of the infectious agent [4].

The prevalence study may some way beneficial to check the infection among the susceptible population. Furthermore, the epidemiology regarding CPV in the study area is very scarce. Therefore, the present study was conducted to determine the prevalence of CPV infection in the Bhubaneswar city of Odisha, for which effective control measures could be carried out.

## Materials and Methods

### Ethical approval

Ethical approval was not necessary. All the animals under this research were clinical cases and were examined, diagnosed and treated as per standard treatment and examination procedure.

### Study area

**Figure-1:**
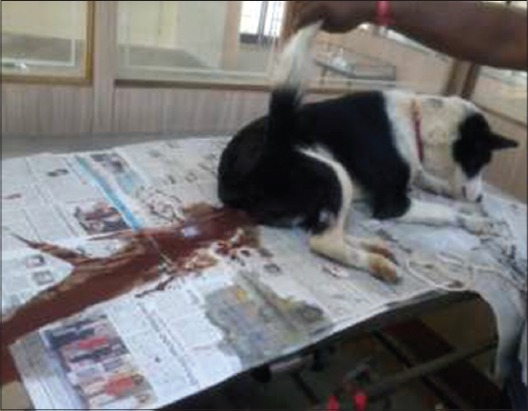
Dog showing bloody diarrhea.

Dogs in and around Bhubaneswar suffering from diarrhea and vomiting were selected for the study as the suspected cases of parvovirus infection (Figure-1). Suspected cases were mostly presented for treatment to Teaching Veterinary Clinical Complex, College of Veterinary Science and Animal Husbandry, OUAT, Odisha from different parts of Bhubaneswar.

### Sample collection

A total of 71 dogs with a history of vomiting and diarrhea were taken in the study for collection of samples during the period of December 2012 to March 2013. They were of different age groups, different breeds and of both genders. In the above study, dogs were categorized into different age groups such as between 1 and 3 months, 3-6 months, 6-12 months and above 12 months. All data regarding age, breed and sex of the suspected dogs were recorded.

Fecal samples were collected from the suspected dogs by introducing a swab into the anus of diarrheic dogs and homogenized (10% w/v) in sterile phosphate buffer saline (PBS). The samples were kept in 2 ml collection tube with the swab. After thorough mixing of the sample in PBS, the swab was removed by squeezing on the inner surface of the collection tube. Then samples were stored at −20°C until further use.

### Preparation of fecal samples

The fecal suspensions with PBS were processed by centrifuging at 1500 xg for 10 min at 4°C. Then supernatants were collected in 2 ml collection tube. The supernatant (500 µl) was boiled for 10 min at 100°C to inactivate the polymerase chain reaction (PCR) inhibitors and chilled on ice as described previously [5]. After boiling the sample was centrifuged at 1500 xg for 5 min and the collected supernatant was used as a source of DNA template for PCR. The lysate was stored at −20°C until further use. The commercial vaccine against CPV, Canigen DHPPi (Virbac) was used as positive control after a pretreatment by fast boiling method.

### PCR

A reaction volume of 25 µl was prepared, which consisted up 0.5 µl of each forward primer and reverse primer (IDT, 10 pico mole concentration) (555 forward- 5’-CAGGAAGATATCCAGAAGGA-3’ and 555 reverse- 5’-GGTGCTAGTTGATATGTAATAAACA-3’ primers used by Buonavoglia *et al*. [6]), 0.5 µl of 2 mM dNTPS (Fermentas), 2.5 µl of Taq DNA buffer A 10X (tris with 15 mM of MgCl_2_) (Genei, Bangalore, India), 0.25 µl of *Taq* DNA polymerase (5 U/µl concentration, Genei, Bangalore) and finally 5 µl of lysate was added. Then the rest volume was adjusted to 25 µl by addition of nuclease-free water (Genei, Bangalore).

The reaction mixture was prepared in 200 µl PCR tubes (Axygen). The amplification was performed in a thermocycler (Applied Biosystems) with a reaction condition comprised of an initial denaturation at 94°C for 2 min, then 40 cycles of denaturation at 94°C for 45 s, primer annealing at 61°C for 45 s and extension at 72°C for 2 min and a final extension at 72°C for 10 min. The amplified PCR products were analyzed on 1% agarose gel (Lonza, USA) with the positive control and visualized under UV transilluminator (Alpha Innotech, USA).

### Epidemiological analysis

Positive cases were analyzed as per different breeds, age groups and sexes in order to conduct an epidemiological analysis on the basis of PCR result. The different breeds include Great Dane, German shepherd, Labrador retriever, German spitz, Deshi, Nea politan mastiff, Pug, Golden retriever, Dalmatian, Doberman, Mixed, Chihuahua and Rottweiler. The different breeds were classified under four groups such as Group-1 (1-3 months), Group-2 (3-6 months), Group-3 (6-12) and Group-4 (>12 months). All the data regarding the breed, age and sex were given in the Tables-1-3, respectively.

**Table-1:** Breed-wise incidence of parvovirus infection in dogs.

Breed	PCR positive	PCR negative	Total	Incidence over positive cases (%)	Incidence over total cases (%)
Mixed	3	3	6	10.34	4.22
Rottweiler	2	4	6	6.90	2.82
Deshi	10	15	25	34.48	14.09
Dalmatians	1	2	3	3.45	1.41
German shepherd	5	4	9	17.24	7.04
Doberman	0	3	3	0	0
Nea politan mastiff	1	0	1	3.45	1.41
Chihuahua	0	1	1	0	0
Golden retriever	0	1	1	0	0
Labrador retriever	3	3	6	10.34	4.22
German spitz	2	5	7	6.90	2.82
Pug	1	0	1	3.45	1.41
Great Dane	1	1	2	3.45	1.41
Total	29	42	71	100	40.85

PCR=Polymerase chain reaction

**Table-2:** Age-wise incidence of parvovirus infection in dogs.

Age groups in months	PCR positive	PCR negative	Total case group wise	Incidence over positive cases (%)	Incidence over total cases (%)
1-3	8	12	20	27.59	11.27
3-6	12	10	22	41.37	16.90
6-12	8	16	24	27.59	11.27
>12	1	4	5	3.45	1.41
Total	29	42	71	100	40.85

PCR=Polymerase chain reaction

**Table-3:** Sex-wise incidence of parvovirus infection in dogs.

Sex	PCR positive	PCR negative	Incidence over positive cases (%)	Incidence over total cases (%)
Male	25	25	86.21	35.21
Female	4	17	13.79	5.63
Total	29	42	100	40.85

PCR=Polymerase chain reaction

## Results

**Figure-2:**
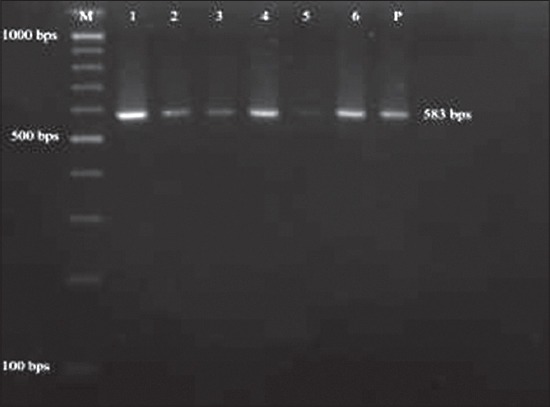
Polymerase chain reaction amplicons of positive samples showing band size of 583 bp. Lane M: Molecular weight marker (100 bp Gene Ruler ladder). Lane 1-6: Positive samples. P: Positive control.

**Figure-3:**
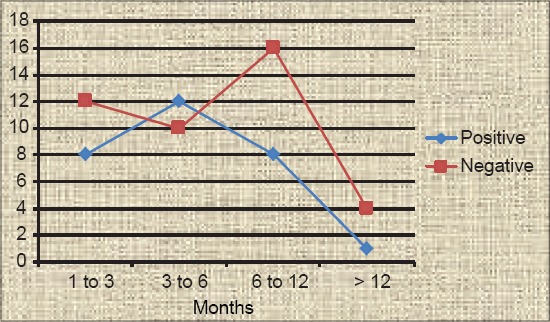
Age-wise distribution of parvovirus infection in dogs.

**Figure-4:**
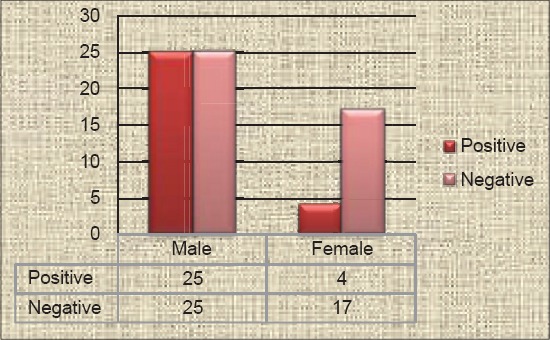
Sex-wise distribution of parvovirus infection in dogs.

The PCR screening of 71 suspected fecal samples revealed 29 (40.85%) samples as positive for canine parvovirus (Figure-2) showing band size of 583 bp using the earlier published primers [6]. The incidence study was calculated with respect to total positive (29) cases as well as total suspected cases (71). The breed-wise incidence rate calculated over positive cases was found to be higher in Deshi/local breeds (34.48%), followed by German shepherd (17.24%), equal incidence in mixed and Labrador retriever (10.34%), Rottweiler and German spitz showed 6.9% each and lower incidences in four breeds (3.45%) such as Dalmatians, Nea politan mastiff, Pug and Great Dane (Table-1). Similarly incidence rate over total screened cases was found to be the highest in Deshi breeds (14.09%) and lower incidence of 1.41% is shared equally in four breeds such as Dalmatians, Nea politan mastiff, Pug and Great Dane (Table-1). Age-wise prevalence study over the positive cases revealed the infection being more in the age group of 3-6 months (41.37%), followed by equal incidences in 1-3 months age group and 6-12 months (27.59%), and a low incidence in age groups above 12 months (3.45%) (Table-2). The age-wise distribution pattern was represented in the Figure-3. The incidence was predominantly higher in males (86.21%) in comparison to females (13.79%) with respect to total positive cases (Figure-4), whereas the incidence rate over total suspected cases was found to be 35.21% in males and 5.63% in females (Table-3)

## Discussion

The study revealed an overall prevalence of canine parvovirus infection in suspected dogs to be 40.85% on the basis of PCR assay. In the present study, a low incidence rate was observed as other cases screened positive for hookworm infection. The above finding was in accordance with those of Phukan *et al*. [7] to some extent, who reported 42.29% incidence of CPV infection among suspected dogs by sandwich ELISA method in Assam, India. Srinivas *et al*. [8] also reported 53.90% incidence by PCR assay using H primer in five states of southern India. However, higher incidences were reported by Phukan *et al*. [9] such as 64% positive by sandwich ELISA and 76% by indirect ELISA. Singh *et al*. [10] reported a higher incidence of 63%. Such high incidence might be due to prevalence of endemic infection in the population under study. In contrast Joshi *et al*. [11] reported lower incidences up to 23.02% by counter immunoelectrophoresis, which might be due to the less sensitivity of detection method. However, these variations observed in the prevalence are difficult to explain due to the different study area and difference in the methods of sample analysis. Breed-wise distribution of CPV revealed Deshi/local breeds (34.48%) were more prone to this infection than that of exotic breeds. Similar findings were reported previously [12] where 27.23% incidence was observed in the local breed. Shukla *et al*. [13] also reported higher prevalence of 56.9% in nondescript dogs and a lower prevalence of 20% in Dalmatian breed. More incidences in Deshi breeds might be due higher population density of this breed making their close proximity to spread the infection or poor vaccination schedule being followed by the owners of Deshi breed due to lack of awareness among them. No specific comments can be made on breed susceptibility as the population density of breeds varies from one geographical area to another [13]. Among the exotic breeds, German shepherd and Labrador retriever were found to be more susceptible with incidences of 17.24% and 10.34%, respectively. It was reported earlier that medium and large breeds are more susceptible to CPV infection [14-16]. Therefore, it might be a reason for a higher incidence in German shepherd and Labrador retriever. Singh *et al*. [10] reported breed wise prevalence of CPV was highest in Doberman (77.78%), followed by Spitz (78.57%), German shepherd (70.00%), Labrador (68.75%), Pomeranian (45.45%). Mixed breeds were also susceptible to infection at the same rate as that of Labrador retriever, followed by lower incidences in other breeds such as Rottweiler, Spitz, Dalmatian, Pug, Great Dane, Nea politan mastiff. These breeds are rarely kept by pet owners; hence, this might be a reason for the lower incidence. Age-wise prevalence was found to be more between the age group of 3-6 months (below 6 months), followed by age group of 1-3 months, 6-12 months and the least above 12 months. This higher prevalence rate below 6 months also reported earlier [8,17-19]. The higher incidence of CPV below 6 months might be due to the affinity of the virus for rapidly multiplying intestinal crypt cells in weaning pups with higher mitotic index due to changes in bacterial flora as well as in the diet due to weaning [20,21]. The fall in maternal antibody level after 3 months age might be one of the predisposing factors, which make the age group of 3-6 months old more prone to CPV and as they advance in age become prone to the infection in endemic areas due to decline in protective titers [12,21]. Above 1 year age, very less incidences were recorded, which might be possible due to developed antibody in the adults either due to vaccination schedule practiced or due to mild exposure to virus leading to build up antibody in the host or some other reasons that need to be explored. In contrast Phukan *et al*. [7] reported highest incidence in the age group of 7-12 months followed by 1-6 month, 13 months and above. The prevalence in the age of 7-12 month might be due to improper age or timing of vaccination, non-boostering of the animals and improper maintenance of cold chain for storage of vaccines [22]. We found that males are mostly susceptible to parvovirus infection than that of females. Of 29 positive cases, 25 (86.21%) were males, and 4 were females (13.79%). The present finding was corroborated with Gombac *et al*. [23]. Thomas *et al*. [24] also reported a higher incidence of CPV (78.26%) in male dogs. Reason behind this high incidence in males may be because most of the admitted dogs (70.42%) were male [24]. However again it can be explained that as male dogs are mostly suffering from an infection, for which more male dogs were being admitted for treatment. The higher incidence in males was also reported by Phukan *et al*. [7], Srinivas *et al*. [8], Tajpara *et al*. [12], Deka *et al*. [20] and Thomas *et al*. [24]. However, some workers reported that there was no influence of sex on the incidence of CPV [25,26]. The high prevalence of CPV in male dogs might be due to more chance of exposure due to certain behavioral pattern and selective preference of keeping male dogs by pet owners [20,27].

## Conclusion

The PCR screening of 71 suspected fecal samples revealed 29 samples positive for CPV with an incidence of 40.85% in the area of study. The epidemiological analysis revealed that Deshi breeds are found to be at a higher risk of getting this infection compared to others, along with age susceptibility being more below 6 months and the sex-wise incidence found to be more in male dogs.

## Authors’ Contributions

MB, SKP, PKS, APA and RCP designed the experiments. MB, SD and SP carried out the experimental work. SKP, PKS and MB were involved in scientific discussion and analysis of the data. MB, SKP, PKS and APA drafted and revised the manuscript. All authors read and approved the final manuscript.

## References

[1] Carmichael L (2003). Canine infectious diseases- A personal perspective. Proceedings in the international symposium on “Reunion Mundial de Lideres en la Education Veterinaria” that commemorated the 150th anniversary of veterinary education in the college of veterinary medicine, National autonomous university of Mexico: August 16th 2003, at D.F. Baker Institute for Animal Health.

[2] Bargujar J, Ahuja A, Bihani D.K, Kataria N, Dhuria D (2011). Studies on prevalence, clinical manifestations and therapeutic management in dogs suffering from canine parvovirus infection. J. Canine Dev. Res.

[3] Balu P.A, Thangaraj T.M (1981). Canine viral gastroenteritis a clinical report. Indian J. Vet. Med.

[4] Nandi S, Kumar M, Chidri S, Chauhan R.S (2008). Current status of canine parvo virus infection in dogs in India and its pathogenesis. Indian J. Vet. Pathol.

[5] Schunck B, Kraft W, Truyen U (1995). A simple touch-down polymerase chain reaction for the detection of canine parvovirus and feline panleukopenia virus in faeces. J. Virol. Methods.

[6] Buonavoglia C, Martella V, Pratelli A, Tempesta M, Cavalli A, Buonavoglia D, Bozzo G, Elia G, Decaro N, Carmichael L.E (2001). Evidence for evolution of canine parvovirus type-2 in Italy. J. Gen. Virol.

[7] Phukan A, Baishya B, Deka D, Boro P.K (2010). Prevalence of canine parvovirus infection in Assam. Indian Vet. J.

[8] Srinivas V.M.V, Mukhopadhyay H.K, Antony P.X, Pillai R.M (2013). Molecular epidemiology of canine parvovirus in Southern India. Vet. World.

[9] Phukan A, Sarma D.K, Deka D, Boro P (2005). Standardization of ELISA for detection of canine parvovirus infection. Indian Vet. J.

[10] Singh D, Verma A. K, Kumar A, Srivastava M, Singh S. K, Tripathi A. K, Srivastava A, Ahmed I (2013). Detection of canine parvo virus by polymerase chain reaction assay and its prevalence in dogs in and around Mathura, Uttar Pradesh, India. Am J. Biochem. Mol. Biol.

[11] Joshi D.V, Singh S.P, Rao V.D.P, Patel B.J (2000). Diagnosis of canine parvovirus infection by counter immunoelectrophoresis. Indian Vet. J.

[12] Tajpara M.M, Jhala M.K, Rank D.N, Joshi C.G (2009). Incidence of canine parvovirus in diarrhoeic dogs by polymerase chain reaction. Indian Vet. J.

[13] Archana Shukla P.C, Gupta D.K, Kumar B (2009). Epidemiology on canine parvovirus infection. Indian J. Vet. Res.

[14] Sellon R.K, Ettinger S.J, Feldman E.C (2005). Canine virus disease. Veterinary Internal Medicine.

[15] McCaw D.L, Hoskins J.D, Greene C.E (2006). Canine viral enteritis. Infectious Diseases of the Dog and Cat.

[16] Morais M.P, Costa P.R, Flores E.F (2007). Parvoviridae. Virologia Veterinária.

[17] Mohanraj J, Mukhopadhyay H.K, Thanislass J, Antony P.X, Pillai R.M (2010). Isolation, molecular characterization and phylogenetic analysis of canine parvovirus. Infect, Genet. Evol.

[18] Xu J, Guo H.C, Wei Y.Q, Shu L, Wang J, Li J.S, Cao S.Z, Sun S.Q (2013). Phylogenetic analysis of canine parvovirus isolates from Sichuan and Gansu Provinces of China in 2011. Transbound Emerg. Dis.

[19] Parthiban S, Mukhopadhyay H.K, Panneer D, Antony P.X, Pillai R.M (2011). Isolation and typing of canine parvovirus in CRFK cell line in Puducherry, South India. Indian J. Microbiol.

[20] Deka D, Phukan A, Sarma D.K (2013). Epidemiology of parvovirus and coronavirus infections in dogs in Assam. Indian Vet. J.

[21] Stepita M.E, Bain M.J, Kass P.H (2013). Frequency of CPV infection in vaccinated puppies that attended puppy socialization classes. J. Am. Anim. Hosp. Assoc.

[22] Carmichael L.E, Joubert J.C, Pollock R.V.H (1983). A modified live canine parvovirus vaccine II immune response. Cornell Vet.

[23] Gombac M, Svara T, Tadic M, Pogacnic M (2008). Retrospective study of canine parvovirosis in Slovenia. Case report. Slovenia Vet. Res.

[24] Thomas J, Singh M, Goswami T.K, Verma S, Badasara S.K (2014). Polymerase chain reaction based epidemiological investigation of canine parvoviral disease in dogs at Bareilly region. Vet. World.

[25] Banja B.K, Sahoo N, Panda H.K, Ray S.K, Das P.K (2002). Epizootiological status of canine viral haemorrhagic gastroenteritis in Bhubaneswar city. Indian Vet. J.

[26] Sanjukta R, Kumar M, Mandakini R (2011). Epidemiological study of canine parvovirus. Indian Vet. J.

[27] Anderson N.V (1980). Veterinary Gastroenterology.

